# Investigating the Anatomy and Microstructure of the Dentato-rubro-thalamic and Subthalamo-ponto-cerebellar Tracts in Parkinson's Disease

**DOI:** 10.3389/fneur.2022.793693

**Published:** 2022-03-24

**Authors:** Ilona Lipp, Jilu Princy Mole, Leena Subramanian, David E. J. Linden, Claudia Metzler-Baddeley

**Affiliations:** ^1^Cardiff University Brain Research Imaging Centre (CUBRIC), School of Psychology, Cardiff University, Cardiff, United Kingdom; ^2^Division of Psychological Medicine and Clinical Neurosciences (DPMCN), School of Medicine, Cardiff University, Cardiff, United Kingdom; ^3^Department of Neurophysics, Max Planck Institute for Human Cognitive and Brain Sciences, Leipzig, Germany; ^4^Neuroscience and Mental Health Research Institute, Cardiff University, Cardiff, United Kingdom; ^5^School for Mental Health and Neuroscience, Faculty of Health, Medicine and Life Sciences, Maastricht University, Maastricht, Netherlands

**Keywords:** Parkinson's disease, basal ganglia, cerebellum, cortico-cerebellar pathways, diffusion tractography, dentato-rubro-thalamic tract, subthalamo-ponto-cerebellar tract, microstructure

## Abstract

Cerebellar-thalamic connections play a central role in deep brain stimulation-based treatment of tremor syndromes. Here, we used diffusion Magnetic Resonance Imaging (MRI) tractography to delineate the main cerebellar peduncles as well as two main white matter tracts that connect the cerebellum with the thalamus, the dentato-rubro-thalamic tract (DRTT) and the subthalamo-ponto-cerebellar tract (SPCT). We first developed a reconstruction protocol in young healthy adults with high-resolution diffusion imaging data and then demonstrate feasibility of transferring this protocol to clinical studies using standard diffusion MRI data from a cohort of patients with Parkinson's disease (PD) and their matched healthy controls. The tracts obtained closely corresponded to the previously described anatomical pathways and features of the DRTT and the SPCT. Second, we investigated the microstructure of these tracts with fractional anisotropy (FA), radial diffusivity (RD), and hindrance modulated orientational anisotropy (HMOA) in patients with PD and healthy controls. By reducing dimensionality of both the microstructural metrics and the investigated cerebellar and cerebellar-thalamic tracts using principal component analyses, we found global differences between patients with PD and controls, suggestive of higher fractional anisotropy, lower radial diffusivity, and higher hindrance modulated orientational anisotropy in patients. However, separate analyses for each of the tracts did not yield any significant differences. Our findings contribute to the characterization of the distinct anatomical connections between the cerebellum and the diencephalon. Microstructural differences between patients and controls in the cerebellar pathways suggest involvement of these structures in PD, complementing previous functional and diffusion imaging studies.

## Introduction

The main pathophysiological feature of Parkinson's disease (PD) is the loss of substantia nigra dopaminergic neurons of the basal ganglia (1). This loss affects subcortical structures, such as the putamen and the thalamus, which are connected to regions of the cortex such as the Supplementary Motor Area (SMA) and the motor cortex (2). Accumulating evidence from anatomical studies in animals and functional studies in humans shows the influence of the cerebellum in Parkinsonian motor symptoms such as akinesia/rigidity, tremor, dyskinesia, gait disturbances, and some nonmotor symptoms such as cognition and olfaction (3). Motor symptoms of PD only arise after 80% of dopamine in the striatum is depleted and compensatory processes have been suggested to occur within and outside the basal ganglia. These should be reflected in the connectivity between motor cortex, basal ganglia, and thalamus (4).

Parkinson's disease also affects white matter anatomy within the cerebellum such as the cerebellar peduncles. Previous studies have found that size and microstructural features of the main cerebellar peduncles can help in differentiating PD from other conditions such as essential tremor, multiple system atrophy, and progressive supranuclear palsy (5–9). Additionally, primate models of PD suggest that the degeneration of nigrostriatal dopaminergic neurons may have direct effects on basal ganglia-thalamic and cerebellar-thalamic pathways (10).

Two pathways that facilitate the communication between the cerebellum and the basal ganglia networks and, hence, may be important in PD are the subthalamo-ponto-cerebellar tract (SPCT) and the dentato-rubro-thalamic tract (DRTT). The SPCT carries the basal ganglia output to the cerebellum. It originates in the subthalamic nucleus, which is a deep brain stimulation target (11) and travels through the pons into the contralateral cerebellar cortex (12). Little is known about the specific functions of the SPCT but because it seems to be one of the main input pathways toward the cerebellum and connects it with input from the subthalamic nucleus (and, by extension, the hyperdirect pathway from cortex), a role in motor control is likely.

In contrast, the DRTT has been described as an ascending tract carrying the main efferent pathways from the cerebellum to the thalamus (13). It ascends from the dentate nucleus through the superior cerebellar peduncle toward the red nucleus and decussates in the midbrain to reach the thalamus (14, 15). A fraction of the fibers has been shown to run ipsilateral, having led to the introduction of the terms “decussating DRTT” vs. “nondecussating DRTT” (16, 17).

The DRTT plays an important role for deep brain stimulation of movement disorders, as it connects to specific nuclei of the thalamus involved in motor pathways, such as the ventral intermediate nucleus of the thalamus, which is used as a surgical target for essential tremor (18–22) and other tremor syndromes (23). Deep brain stimulation-induced reduction of tremor may be a result of altering the functional coupling between cerebellum and thalamus that is mediated by the DRTT (17). Therefore, substantial effort has been put into *in-vivo* reconstruction of this tract (19, 24–29).

Diffusion MRI tractography is presently the only technique that allows for *in-vivo* and non-invasive investigation of white matter connections in humans and their role in health and disease. A specific challenge related to studying the DRTT and the SPCT with diffusion tractography is that the tracts are thin, long, multisynaptic, and cross over to the contralateral hemisphere, making them prone to crossing fiber artifacts. Diffusion tensor imaging-based tracking cannot resolve principal fiber direction in areas of crossing/touching fibers and is, therefore, prone to false negatives in these tracts (29). Here, we applied high angular resolution diffusion-weighted imaging (HARDI) (30) together with a damped Richardson–Lucy (dRL) spherical deconvolution-based tractography algorithm (31) to allow the recovery of multiple fiber orientations within each voxel. Given the well-described anatomical trajectories of the cerebellar peduncles and the DRTT and the SPCT, at least from cerebellum to (sub)thalamic regions, we employed deterministic tractography in order to detect and use all the streamlines that followed the assumed path.

Here, we first developed a delineation protocol in a 60-direction HARDI dataset from a group of young healthy participants (YHPs) and then applied it to reconstruct the DRTT and the SPCT in data from individuals with PD and age-matched healthy controls (MHCs) that were acquired with a standard clinical 30 directions diffusion imaging protocol. We then investigated the microstructural differences between patients and controls in the DRTT and the SPCT as well as in the cerebellar peduncles.

## Materials and Methods

### Sample 1: Young Healthy Participants

The development of the tractography protocols for the DRTT and the SPCT tracts was based on baseline data from 20 healthy young adults recruited for a study into cognitive training [(32, 33); Table 1].

**Table 1:** Demographics of the young healthy participants (YHPs), patients with Parkinson's disease (PD), and matched healthy controls (MHCs).

	**YHP (*n* = 20)**	**PD (*n* = 24)**	**MHC (*n* = 25)**	**Differences between PD and MHC**
Age	25.4 ± 4.84	63.42 ± 10.82	64.84 ± 8.22	*p* = 0.61 Two sample *t*-test
Sex (M:F)	12:8	22:2	16:9	*p* = 0.02, Chi squared test
No of diffusion directions	60	30	30	-
H and Y stage	NA	1.75 ± 0.47	NA	-
MOCA	NA	26.54 ± 2.01	NA	-
LEDD (mg)	NA	537.64 ± 340.69	NA	-

*MOCA, The Montreal Cognitive Assessment; H and Y, Hoehn and Yahr Stage; LEDD, Levodopa Equivalent Daily Dose; YHP, Young Healthy Participants; PD, Parkinson's disease patients; HC, Healthy Controls; M, Male; F, Female; R, Right; L, Left*.

### Sample 2: Patients With PD and MHCs

This study made use of the MRI data from twenty-four patients with PD in the Hoehn and Yahr stages I–III of the disease and twenty-six age-MHCs. Patients with PD were recruited from National Health Service (NHS) clinics in South Wales for an intervention study into neurofeedback and exercise (34) and the baseline data of this study were analyzed here. Patients' medication and the procedure for cognitive testing are described elsewhere (34). Age- and sex-MHC participants were recruited for a larger study into aging (35). Note that this is the same cohort of patients and controls that were reported in a previous study (36). All the participants' characteristics are shown in Table 1.

The patient study was approved by the local NHS research ethics committee. The studies involving healthy controls were approved by the ethics committee of the School of Psychology at Cardiff University. All the participants provided a written informed consent.

### Magnetic Resonance Imaging Data Acquisition

Magnetic resonance imaging data were acquired for all the participants on a 3T General Electric (GE) Signa HDx System, using an eight channel receive-only head radiofrequency (RF) coil. T1-weighted structural scans were acquired using a three-dimensional (3D) fast spoiled gradient recalled sequence (FSPGR) with a slice thickness of 1 mm, acquisition matrix: 256 × 256, voxel size: 1.0 mm^3^ × 1.0 mm^3^ × 1.0 mm^3^, Echo time (TE) = 3 ms, Repetition time (TR) = 7.9 ms, 172 slices (178 sliced for healthy young controls from training study), and total acquisition time of 7 min. Diffusion-weighted MRI data were acquired using a peripherally gated twice-refocused pulse-gradient spin-echo echo-planar imaging (EPI) sequence providing whole oblique axial (parallel to the commissural plane) brain coverage. Data were acquired with parallel imaging [Array coil Spatial Sensitivity Encoding (ASSET) factor = 2] from 60 axial slices of 2.4 mm thickness, with a field of view of 23 cm, and an acquisition matrix of 96 × 96. Diffusion encoding gradients (b = 1,200 s/mm^2^) were applied along 60 (for the YHPs) or 30 (for patients with PD and the MHCs) isotropically-distributed directions using an optimized gradient vector scheme (37). Three or six nondiffusion-weighted images were acquired for the 30 or 60 directions, respectively. The 60 directions scan was cardiac gated and took approximately 30 min, while the 30 directions scan took a total acquisition time of 15 min.

### Diffusion MRI Data Processing

The diffusion-weighted data were corrected for head motion, distortions induced by the diffusion-weighted gradients, and EPI-induced geometrical distortions by registering each diffusion image to their respective T1-weighted anatomical image (38) with appropriate reorientation of the encoding vectors in ExploreDTI [version 4.8.3; (39)].

### Deterministic Tractography

Deterministic tractography was used to reconstruct the fiber tracts of interest. Whole-brain tractography was performed for each data set in native space using the dRL spherical deconvolution-based algorithm (31), which allows the recovery of multiple fiber orientations within each voxel, including in those voxels affected by cerebrospinal fluid partial volume. The dRL tracking algorithm estimated peaks in the fiber orientation density function (fODF) using each voxel as a seed point and propagated in 0.5 mm steps along these axes. The fODF peaks were re-estimated at each new location (40). If the fODF threshold fall below 0.05 or the direction of pathways changed through an angle > 45° between successive 0.5 mm steps, tracking was terminated. The same procedure was then repeated by tracking in the opposite direction from the initial seed point.

Three-dimensional fiber reconstructions of the cerebellar and basal ganglia-cerebellar white matter pathways were achieved by applying waypoint region of interest (ROI) gates based on Boolean logic operators (such as AND and NOT) to isolate specific pathways from the whole-brain tractography data. ROIs were drawn manually on color-coded fiber orientation maps (41, 42) in native space guided by the following anatomical landmark protocols. Each reconstructed tract was visually inspected and any obvious outlier streamlines that were not consistent with their known anatomy were excluded by drawing “NOT” regions.

### Protocols for Reconstruction

#### Dentato-rubro-thalamic Tract (DRTT)

**Figure 1:**
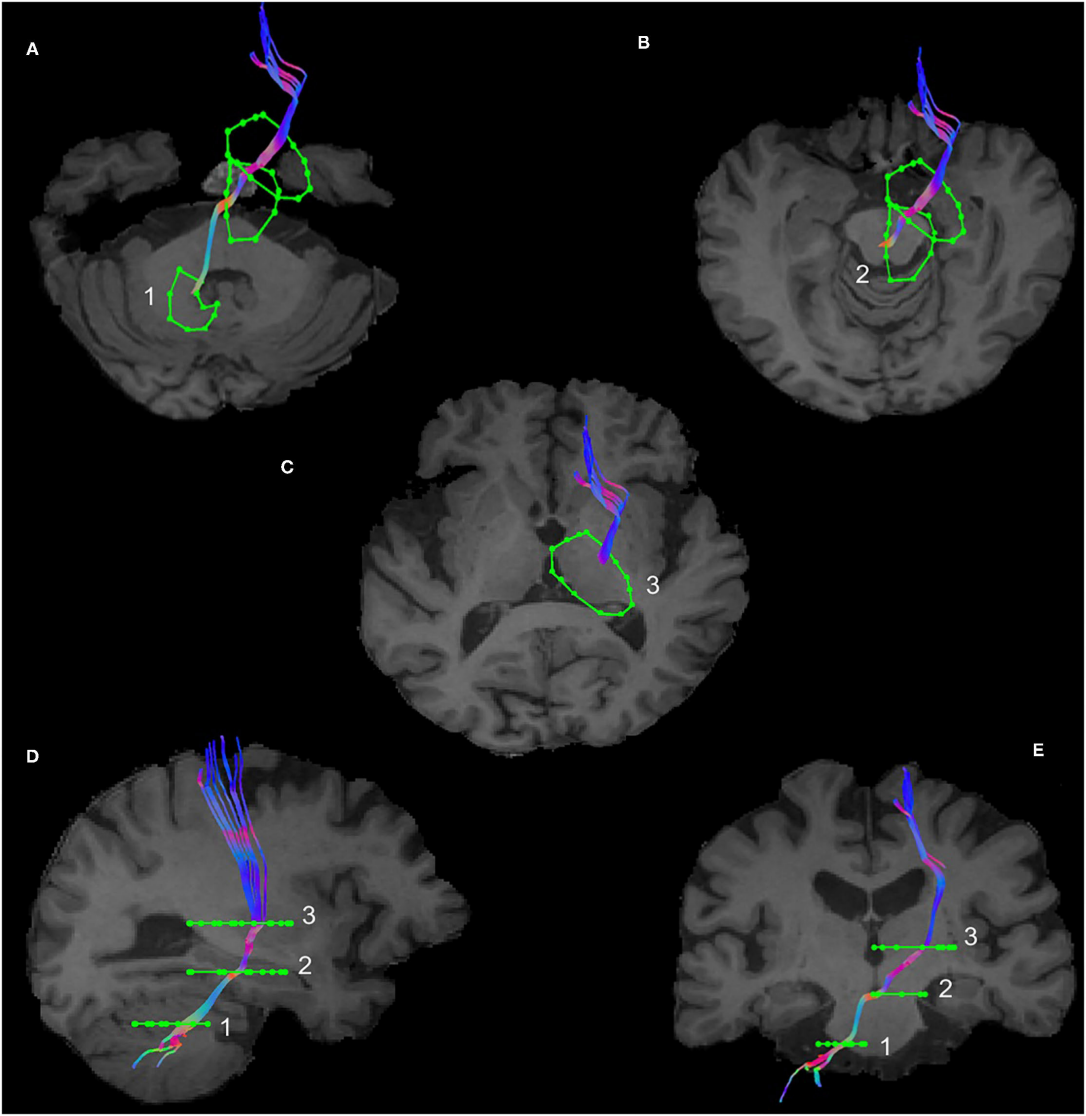
The dentato-rubro-thalamic tract (DRTT) tract along with the regions of interest (ROIs) in axial, sagittal, and coronal views of T1 anatomical scan in an example data set. **(A)** Visualizes the DRTT tract from the dentate nucleus, **(B)** shows the DRTT decussating in the midbrain, and **(C)** shows the DRTT passing through the thalamus in the axial view. **(D)** Shows the entire DRTT with its cortical connections on a sagittal view and **(E)** shows the entire DRTT with its cortical connections on a coronal view.

The dentate nucleus was identified in the axial view of the brain inferolateral to the fourth ventricle, where the temporal lobe was still visible and the slice cuts through the middle of the basilar pons and an ROI was drawn as shown in Figure 1A. The thalamus was identified in the axial view and a second ROI was drawn contralateral to the AND region of the dentate nucleus as shown in Figure 1C. The red nucleus is located in the midbrain, inferomedial to the thalamus, inferolateral to the 3rd ventricle, and posterior to the cerebral peduncle (16). The decussation point of the DRTT was identified along with the red nucleus and the substantia nigra in the diffusion principal direction color-coded image and another ROI was drawn (Figure 1B). This procedure was repeated for the other hemisphere and for all the participants. Our reconstruction protocol, therefore, only considered the decussating part of the DRTT, which is a commonly used approach (20). The full DRTT of a representative participant is shown in Figures 1D,E and the right and left DRTTs are shown in Supplementary Figure 1.

#### Subthalamo-ponto-cerebellar Tract (SPCT)

**Figure 2:**
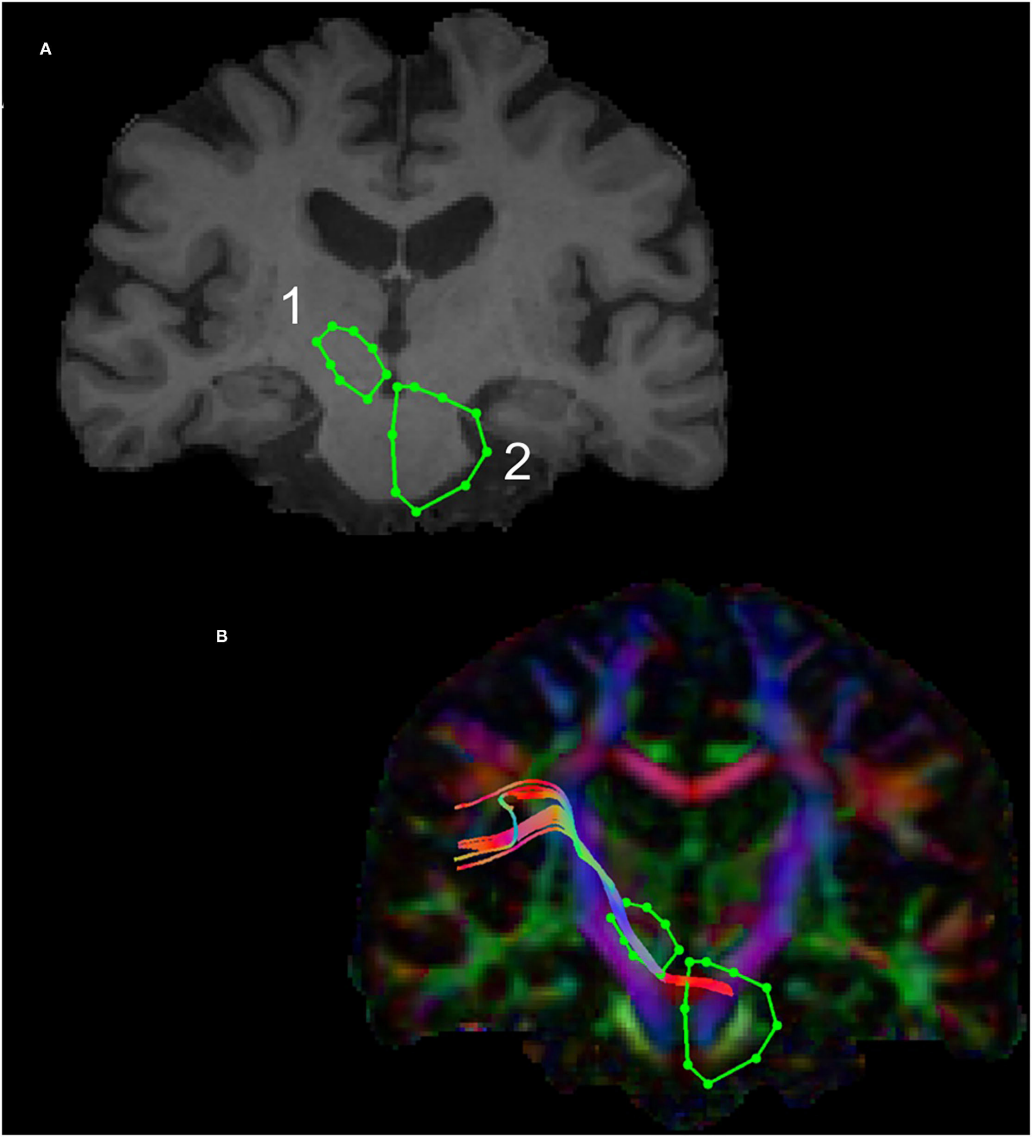
Region of Interest(s) (ROIs) and reconstruction of the subthalamo-ponto-cerebellar tract (SPCT) in an example dataset in coronal view in T1 anatomical **(A)** and diffusion color-coded image **(B)**. The first ROI (1) is drawn around the subthalamic nucleus and the second ROI (2) is drawn around the contralateral pons region.

**Figure 3:**
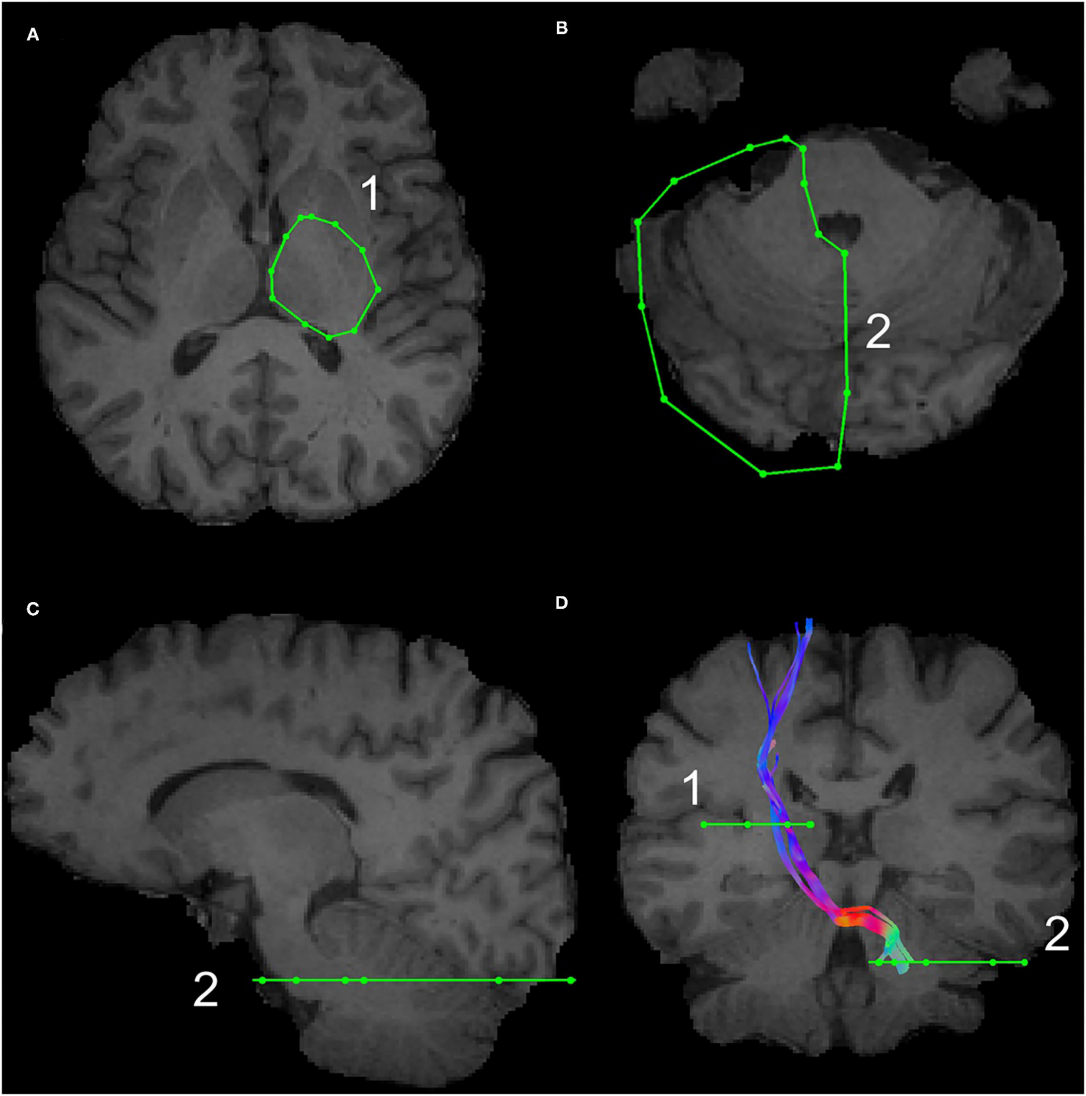
The alternative Region of Interest(s) (ROIs) used for reconstruction of the DRTT and the SPCT. The ROI around the thalamus covering the internal capsule is shown in **(A)**, the ROI covering the contralateral cerebellar peduncle is shown in **(B,C)**. An example of the SPCT obtained using this alternative method is shown in **(D)**.

The subthalamic nucleus is a small biconvex-shaped nucleus modulating the basal ganglia output and is located inferior to the thalamus and superior to the substantia nigra and lateral to the internal capsule (43). For the reconstruction of the SPCT, an ROI was drawn on a coronal plane around the subthalamic nucleus as shown in Figure 2. The second ROI was drawn around the contralateral pons region surrounding the decussation of the SPCT into the cerebellar hemispheres (Figure 2). This procedure was repeated for the other hemisphere and for all the participants. For some datasets, the methods described above were unsuccessful in delineating the SPCT or the DRTT due to difficulties in identifying the above described anatomical landmarks. If this was the case, a different approach was taken: an ROI was drawn around the thalamus covering the posterior limb of the internal capsule in an axial view of the brain (Figure 3A) and a second ROI around the contralateral cerebellar hemisphere just below the pons was drawn also in the axial view of the brain as shown in Figures 3B,C. Appropriate NOT ROIs for fiber exclusion were drawn to delineate the DRTT and the SPCT separately. A representative SPCT obtained using this alternative method is shown in Figure 3D. The right and left SPCTs are also shown in Supplementary Figure 2.

Additionally, the main cerebellar tracts [middle cerebellar peduncle (MCP), inferior cerebellar peduncles (ICP), and superior cerebellar peduncle (SCP)] were described according to the previous descriptions (44, 45).

#### Middle Cerebellar Peduncle

For the reconstruction of the MCP, AND regions were drawn around the pons on a coronal slice on both the hemispheres as shown in Supplementary Figure S3A. Appropriate NOT regions were drawn to eliminate any erroneous fibers. The MCP in an example dataset on the axial and sagittal slices of a T1 anatomical image is shown in Supplementary Figures S3B, and C, respectively.

#### Superior Cerebellar Peduncle

The SCP was identified in the coronal plane of a color-coded fiber orientation image and the AND region was drawn around the SCP (visible as a small light blue structure) as shown in Supplementary Figure S4A. Additionally, two large NOT gates were drawn above (Supplementary Figure S4, red ROI 1) and in front of the fornix (Supplementary Figure S4, red ROI 2) to exclude tracts going to the frontal and cortical regions. Two more NOT regions were drawn, one NOT region at longitudinal fissure to cut fibers that cross into the other hemisphere (Supplementary Figure S4, red ROI 3) and another to cut fibers that go into the brainstem (Supplementary Figure S4, red ROI 4). This procedure was repeated for both the hemispheres separately. The SCP in an example dataset in the sagittal and coronal views is shown in Supplementary Figures S4A and B, respectively.

#### Inferior Cerebellar Peduncle

For the reconstruction of the ICP, the large NOT region (red ROI 1, ROI 2, and ROI 3) from the SCP reconstruction were retained. In a color-coded fiber orientation image, an AND region was drawn in the axial slice around the ICP (visible as the small light blue structure) located by moving up slices from the bottom of the brain where the medial lemniscus and cerebellar peduncle are visible (Supplementary Figure S5A). Another AND gate was drawn around the region (visible as the light green structure) located at approximately five slices above the first AND gate (Supplementary Figure S5B). The ICP ROI placement used here was previously described in Catani et al. (46). The ICP of a representative participant in sagittal views of a color-coded fiber orientation image and a T1 image is shown in Supplementary Figures S5C, D, respectively.

#### Interoperator Reliability of Tract Reconstruction

Tracts for all the participants were reconstructed by JPM. In order to assess the reliability and reproducibility of the reconstructions across researchers, all the tracts were independently reconstructed by a second operator (IL) for a subset of randomly selected participants (5 from each group). Reliability was assessed by calculating overlap dice coefficient scores using the formula: 2 × number of overlapping voxels/(number of voxels in tract by operator 1 + number of voxels in tract by operator 2) and subsequently converted to percentage units.

#### Tractometry

For individual tracts, we calculated the microstructural parameters of fractional anisotropy (FA) and radial diffusivity (RD) at each point along the individual streamlines (47) by trilinear interpolation of the surrounding voxels. The means across the tract vertices were then used for statistical analysis. Additionally, hindrance modulated orientational anisotropy (HMOA), was considered as a microstructural measure. The HMOA is defined as the absolute amplitude of the fiber orientation distribution and it provides a fiber population specific index of the diffusion properties along the reconstructed fibers (48). Hence, the HMOA provides more tract specificity. This is important as the cerebellar tracts and the DRTT and the SPCT run in close spatial proximity to each other, occupying partly the same voxels.

The initially reconstructed DRTT and SPCT also included their cortical projections. To include only the portion of the DRTT between the dentate nucleus and the thalamus and the portion of the SPCT from the subthalamic nucleus to the cerebellum for extracting the diffusion metrics, we further segmented them using the Splitter tracts tool within ExploreDTI version 4.8.3. A representative segmented DRTT and SPCT with the ROIs are shown in Supplementary Figure S6.

#### Tract Probability Maps

The probability maps of all the tracts within the brain were mapped out using group-based probability maps for each tract. To create these maps, the Neuroimaging Informatics Technology Initiative (NIfTI)—exported tracts of each participant were registered to the Montreal Neurological Institute (MNI) space. This was done by first registering each participant's structural high-resolution T1-weighted image to the MNI space, using FMRIB Software Library (FSL) FNIRT [FSL version 5.0.9, warp resolution: 10 mm × 10 mm × 10 mm; (49)] and then applying the warp to the tract NIfTI file (which had already been registered to the high-resolution structural scan as part of the preprocessing pipeline in ExploreDTI).

#### Statistical Group Comparison of Tract Microstructure

All the statistical analyses were conducted in the R project for statistical computing [(version 3.6.3) (50)].

Combining microstructural metrics: As the diffusion-based microstructural metrics were highly correlated with each other and the microstructure in the investigated tracts was not expected to be independent, a dimensionality reduction approach was used. The microstructural FA, RD, and HMOA were entered into a principal component analysis (51, 52), using the function *principal* from the *psych* library. Here, the values of all the tracts were vertically concatenated into a matrix with three columns. As not all the tracts could be reconstructed in all the participants, mean imputation was done for missing values (*na.aggregate, zoo* library). When extracting components, we visually inspected Catell's scree plot (53), the eigenvalues of the components (54), as well as the interpretability of the components. Following recommendations to limit the number of extracted components to a minimum (55, 56), only the first component was retained as a general microstructural measure for each tract.

Combining tracts: In a second step, the covariance matrix between all the tracts' component measure was used to perform another principal component analysis to reduce the dimensionality of the tract domain. Based on the same component extraction criteria, again, only one component was retained. The two-sample *t-test* (*stats* library) was then used to compare the component score between patients with PD and controls.

In a second analysis, we explored whether the two groups differed in any microstructural measurements of the tracts. For this, multivariate ANOVAs (MANOVAs) were performed for each tract separately, with FA, RD, and HMOA as dependent variables and group (patients with PD vs. controls) as independent variable. First, multivariate normality was tested, using the Shapiro–Wilk test in the *mvnormtest* library. As for most tracts, the assumption of multivariate normality was violated; the MANOVAs were conducted on ranked data using Munzel and Brunner's method, implemented in the *mulrank* function of the Wilcox' Robust Statistics *(WRS)* library (57). The resulting nine *p*-values were corrected for false discovery rate, using the *p.adjust* function of the *stats* package (58). A test was deemed significant if it had an false discovery rate (FDR)-corrected *p* < 0.05.

## Results

### Proportion of Successful Tract Delineations

The main cerebellar peduncles could be successfully reconstructed in all the participants. The DRTT reconstruction was not successful in all the cases [young healthy controls: left: 16 (80%), right: 15 (75%); patients with PD: left: 17 (71%), right: 16 (67%); MHCs: left: 18 (72%), right: 14 (56%)] neither was the SPCT [young healthy controls: left: 11 (55%), right: 16 (80%); patients with PD: left: 22 (92%), right: 23 (96%); MHCs: left: 19 (76%), right: 19 (76%)]. The finding that the DRTT and the SPCT could not be reconstructed in all the young healthy controls, for which the 60-direction diffusion protocol was used indicates that angular resolution is not the limiting factor for tract reconstruction. From the participants that had undergone the 30-direction protocol, the probability of a successful reconstruction of the left DRTT was unrelated to the probability of a successful reconstruction of the right DRTT (eight participants for which neither could be reconstructed vs. 19 participants for which at least one DRTT could be reconstructed). Results were similar for the SPCT (5 participants for whom neither could be reconstructed vs. eight participants for whom at least one SPCT could be reconstructed). The successful reconstruction of the DRTT was also unrelated to the successful reconstruction of the SPCT. This indicates that failed reconstructions of individual tracts were not related to generally low data quality in these datasets.

### Reliability of Individual Tract Reconstruction

On average, the anatomical overlap between tract reconstructions of the two operators was between 44 and 93% across tracts. Closer inspection revealed that tracts from one operator were consistently larger than tracts from the other operator, whose smaller tracts were mostly comprised within the larger ones. Statistical comparison of the number of voxels transversed by the tracts confirmed this (Supplementary Table 1). The tract-extracted FA values showed strong interoperator correlations, with exception of the values for the SPCT (see Supplementary Table 1).

### Anatomy of the Reconstructed DRTT and SPCT

**Figure 4:**
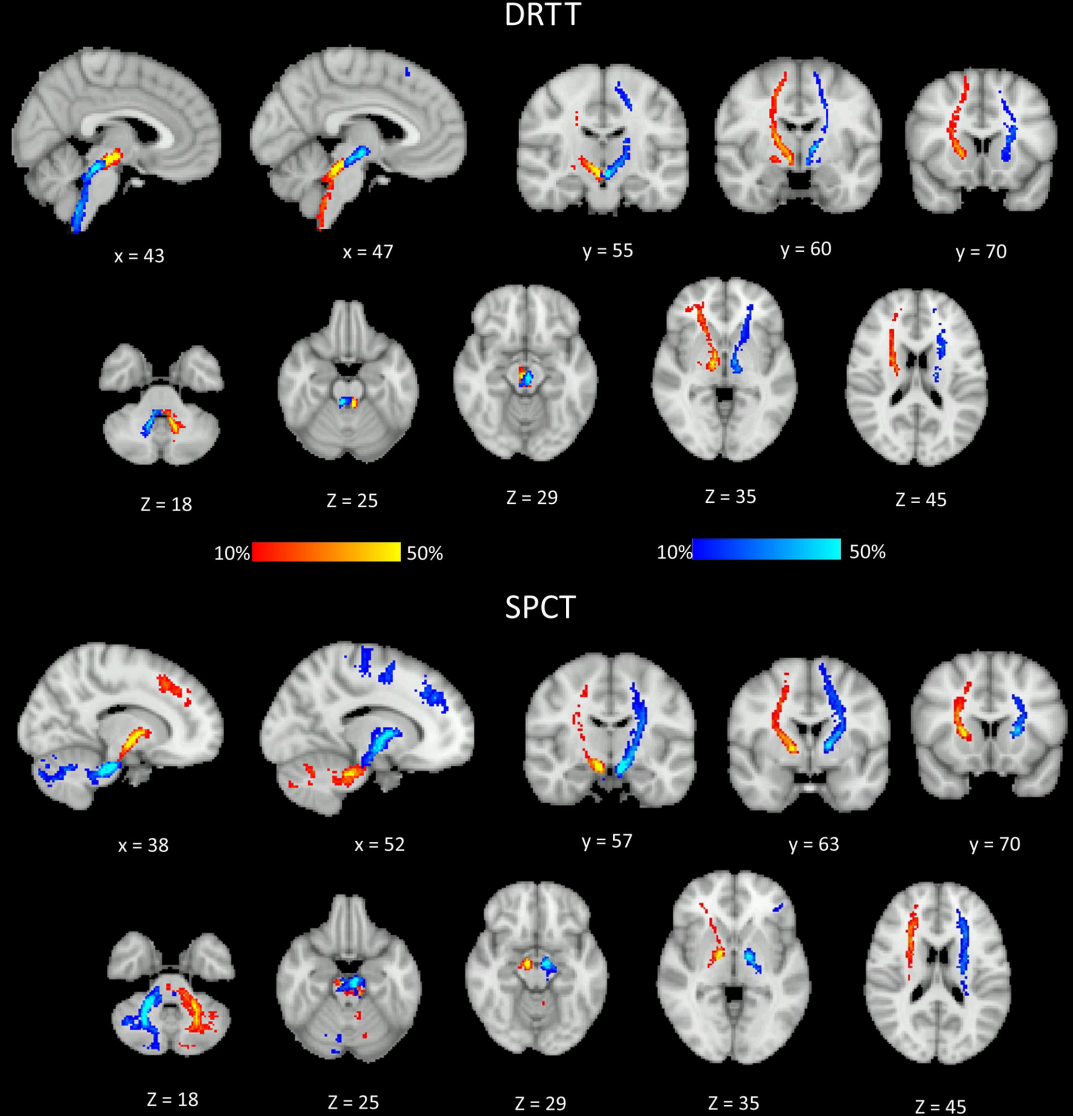
Different sections of the Montreal Neurological Institute (MNI) brain showing the probability (in percent) of the dentato-rubro-thalamic tract (DRTT) and the subthalamo-ponto-cerebellar tract (SPCT) in the Parkinson's disease (PD) group. The display range is set to 10–50%, hence areas of the brain showing yellow and light blue colors have at least 50% of participants' tracts passing through those voxels.

The probability maps depicting the anatomical connections for the DRTT and the SPCT for the PD group are shown in Figure 4. The tract connection maps of the DRTT and the SPCT were visually assessed using the Juelich Histological Atlas, Harvard–Oxford Subcortical Structural Atlas, and the MNI Structural Atlas in both the hemispheres. The DRTT originates from the dentate nucleus and travels superiorly and medially through the SCP toward the midbrain. The DRTT is located medioposteriorly to the corticospinal tract (CST) in the internal capsule and then its cortical connections travel laterally anterior and posterior to the CST. While we did not find any connections between the SPCT and the occipital and temporal lobes, there were connections with the superior parietal lobe.

### Differences Between Patients and Controls

Correlations between the microstructural metrics were high (FA vs. HMOA: *r* = 0.94, FA vs. RD: *r* = −0.73, RD vs. HMOA: *r* = −0.63). Based on the visual inspection of Catell's scree plot (53) and the Kaiser criterion (54), only the first principal component was retained. It explained 85% of the variance and was used as an overall measure of microstructure. FA (0.97) and HMOA (0.94) contributed with a positive loading, whereas RD (−0.84) contributed with a negative loading. The principal component analysis across the tracts yielded a principal component that explained 40% of the variance. All the tracts contributed to this component with a positive loading (Table 2). The two-sample *t*-test revealed higher values for patients with PD than controls [*t*_(38.6)_ = 2.24, *p* = 0.031]. Two other components had an eigenvalue of > 1 (Kaiser's criterion), but the interpretability of these components was limited, even after varimax or oblique rotations (see Table 2, Supplementary Tables 2, 3).

**Table 2:** Principal component analysis on cerebellar tracts.

**#**	**Variance**	**Eigenvalue**	**Tract loadings**	**PD vs. HC**
1	40%	3.61	L DRTT: 0.54, R DRTT: 0.68, L SPCT: 0.32, R SPCT: 0.44, MCP: 0.67, L ICP: 0.66, R ICP: 0.76, L SCP: 0.73, R SCP: 0.78	*t*_(38.6)_ = 2.24, *p* = 0.031
2	16%	1.43	L DRTT: −0.36, R DRTT: 0.18, L SPCT: 0.60, R SPCT: 0.63, MCP: 0.50, L ICP: −0.26, R ICP: −0.21, L SCP: −0.35, R SCP: −0.20	not retained
3	12%	1.04	L DRTT: −0.31, R DRTT: −0.22, L SPCT: 0.49, R SPCT: −0.27, MCP: −0.13, L ICP: 0.58, R ICP: 0.36, L SCP: −0.07, R SCP: −0.31	not retained
4	8%	0.76	Not retained	not retained
5	7&	0.64	Not retained	not retained
6	7%	0.63	Not retained	not retained
7	5%	0.41	Not retained	not retained
8	3%	0.27	Not retained	not retained
9	2%	0.20	Not retained	not retained

*For each resulting component, the explained variance and associated Eigenvalue are reported. Based on Kaiser's criterion, the first 3 components can be retained. For those, the component loadings of all the considered tracts are reported, as is the statistical comparison between patients (PD) and healthy controls (HCs), as calculated with the two-sample t-test. L, Left; R, Right*.

The separate analyses for each tract revealed significant group differences for the right ICP and right SCP, which, however, did not statistically survive the correction for multiple comparisons across tracts (Table 3).

**Table 3:** Results from the MANOVAs for the main cerebellar tracts such as the dentato-rubro-thalamic tract (DRTT) and the subthalamo-ponto-cerebellar (SPCT).

	***N***	*F*	***p***_**uncorr**_	***p***_**corr**_
L DRTT	35	1.72	0.1888	0.2427
R DRTT	30	1.38	0.2497	0.2809
L SPCT	41	2.99	0.0703	0.1809
R SPCT	41	0.07	0.9096	0.9096
MCP	49	1.79	0.1777	0.2427
L ICP	49	2.39	0.1005	0.1809
R ICP	49	4.12	0.0205	0.0923
L SCP	49	2.49	0.0973	0.1809
R SCP	49	5.03	0.0141	0.0923

*Reported are total sample size (N), F statistic, the uncorrected p-value (p_uncorr_), and false discovery rate (FDR)-corrected p-value (p_corr_)*.

## Discussion

In this study, we developed an anatomically guided tractography protocol to reconstruct the DRTT and the SPCT using HARDI data (60 directions) from a group of YHPs. We then applied this anatomical protocol to successfully reconstruct these tracts as well as the main cerebellar peduncles in a standard clinical dataset (30 directions) of patients with PD and MHCs. We further extracted microstructural metrics from all the tracts to investigate differences between patients and controls. Reducing the dimensionality of the tracts with a principal component analysis, we found overall increase in FA, HMOA, and reductions in RD across all the tracts in patients. Individual analysis for each tract separately, however, did not yield any significant group differences.

We were not able to reconstruct the DRTT and the SPCT in all the participants; this could be owed to the specific challenges they pose; for example, they run in close proximity to other tracts and are smaller, longer, and have more curvatures than the main cerebellar peduncles. Such constellation of fibers within imaging voxels can pose challenges for the tracking (59). Residual noise, even though the data were corrected for participant motion, eddy current, and echo planar imaging distortion and air tissue interfaces (24), may have additionally contributed to the failed reconstructions. The choice of the imaging parameters, the image quality, the tracking algorithms, and preprocessing pipelines are also known to affect the quality, precision, and reliability of tractography results (60) and may contribute to false-positive and false-negative findings (61). Additionally, the deterministic tractography approach we used is more conservative than probabilistic tractography (62). However, with probabilistic tractography, the need for an arbitrary threshold would have possibly increased the number of false-positive tracts in our sample. Using anatomical criteria employed on each participant's individual scan, we obtained reproducible tract reconstructions. However, the observation that reconstructions were not always successful for all the datasets highlights the limitations of employed *in-vivo* tractography approach.

The connections of the DRTT followed the previously described animal literature (63) and various studies in humans using tractography (19, 24, 64), histology (14), and *postmortem* dissection studies (12, 14, 15, 24, 27, 65). We successfully replicated the reconstructions of cortical connections of the DRTT from the thalamus in line with previous study: one retrograde transneuronal virus tracer study has found projections from the dentate nucleus and the primary motor, premotor, prefrontal, and posterior parietal areas of the cortex (66). Disynaptic projections from the dentate nucleus to the SMA and pre-SMA were reported by Akkal et al. (67). Another study found that the dentate nucleus projects to and receives input from the primary motor cortex and the dorsolateral prefrontal cortex (68). The dentate nucleus was also found to provide input to the frontal eye field (69). The dentate nucleus projects to the different regions of the prefrontal cortex *via* the thalamus (70). Although some studies have also found that the inferior parietal lobule (71) and posterior parietal cortex (72) are targets for outputs from the dentate nucleus, we did not find any DRTT connections to the parietal cortex in our data.

The connections of the SPCT also followed the findings from nonhuman primates (73). The SPCT originates in the subthalamic nucleus descending through the brainstem and decussating in the upper and/or middle pons, before traveling into the contralateral cerebellar hemisphere. The SPCTs that we reconstructed did not, however, enter the contralateral cerebellar cortex *via* the SCP, as described by the previous study that has described the SPCT in humans (12), but instead through the MCP. The finding of cortical connections of the SPCT from our data is in support of the classical view that cerebellum receives input from widespread neocortical areas including portions of the frontal, parietal, temporal, and occipital lobes (74, 75).

Since our results are based on diffusion tractography, we cannot exclude that some of the cortical projections are in fact different tracts. Due to methodological limitations and challenges, such as the crossing/kissing problem (76, 77), the anatomical accuracy of tractography can be limited. Exact anatomical waypoints can increase accuracy (78). Our reconstruction protocol was anatomically guided, with ROI-defined waypoints of generous size. This minimized the risk of false negatives, but may have led to some false-positive connections, in particular with regard to cortical connections. Additionally, detailed descriptions on the thalamocortical connections of the DRTT and the SPCT are still missing in the literature. It is also noteworthy that the reconstructed tracts do not allow any inferences about the afferent or efferent nature of the connections.

For the comparison between patients and controls, only the parts of the DRTT/SPCT between cerebellum and thalamus/subthalamic nucleus were considered. As correlations between the different microstructural metrics and different tracts were expected and observed, we employed a dimensionality reduction approach before performing statistical comparisons between patients and controls. Patients with PD had a higher value on our global measure of microstructure for the cerebellar tracts, which had a positive loading of FA. This result confirms the direction of our previous findings of increased FA in cortical motor pathways in the same sample (36), although we cannot specifically attribute our global effects of FA. At the level of specific tracts, no group difference remained significant after correction for multiple comparisons.

Previous studies have reported microstructural changes in patients with PD in the cerebellum. One study reported decreased FA in the entire white matter of the cerebellar hemispheres in patients with PD (79). Another study found decreased FA and increased MD in the cerebellum (80). However, at the same time, previous studies have also reported no significant FA alterations in the SCP and the MCP (7, 8, 81–84). In comparison to previous studies, our results indicate increased FA in cerebellar white matter in patients with PD. The increase could point to compensatory mechanisms (85). In particular, functional imaging studies have reported task-specific hyperactivation in the cerebellum during performance of finger sequence tasks and motor learning (86, 87) in PD. Cerebellar hyperactivation has been interpreted as an early compensatory mechanism that seems to decrease, as the disease progresses possibly due to cerebellar degeneration process (3, 87, 88). In a previous study in our cohort (36), we also found increased FA in white matter tracts relevant for motor function. However, it should be noted that we did not find any relationships between interindividual variation in FA and clinical symptoms and/or cognitive and motor functions. An additional consideration concerns the fact that diffusion metrics are sensitive to the underlying fiber configuration and that fiber loss in areas of crossing fibers could lead to increased FA (89).

Another important consideration to make is that the cerebellar involvement may vary according to the various clinical stages of the disease. While in the preclinical stage, the cerebellum might be fully compensating for the loss in basal ganglia function; in later stages, the cerebellar compensation may reduce as the severity of the PD symptoms accumulates (3). Therefore, whether abnormalities in the cerebellar system are found in patients may depend on their individual disease stages. Further studies with larger sample sizes will be needed to address the effect of disease stage on the microstructural abnormalities of cerebellar tracts.

## Conclusion

In this study, we showed that the DRTT and the SPCT can be reconstructed in data of healthy controls and patients with PD, even when acquired with standard clinical diffusion-weighted datasets. However, the reconstruction is not successful in all the individuals and technical advancements will be necessary to improve the sensitivity and specificity of tractography techniques (59). Considering a number of cerebellar tracts in combination, we demonstrated microstructural abnormalities in PD, which may point to compensatory mechanisms at early stages of the disease. Studies in larger patient cohorts, however, are needed to identify the clinical relevance of microstructural changes within individual cerebellar tracts.

## Data Availability Statement

The datasets presented in this article are not readily available because ethical approval to share them was not obtained. Requests to access the datasets should be directed to CM-B (Metzler-BaddeleyC@cardiff.ac.uk) and/or DL (david.linden@maastrichtuniversity.nl).

## Ethics Statement

The studies involving human participants were reviewed and approved by the Ethics Committee of the School of Psychology, Cardiff University, and the local NHS Research Ethics Committee. The patients/participants provided their written informed consent to participate in the research studies.

## Author Contributions

JM, LS, DL, and CM-B: conceptualization. DL and CM-B: supervision and editing. JM and IL: writing and data analysis. JM, IL, DL, and CM-B: methodology. LS and CM-B: data collection. All authors contributed to the article and approved the submitted version.

## Funding

This study received funding from the Wellcome Trust through the Institutional Strategic Support Fund to Cardiff University. The patient data were acquired as part of the study Real-time fMRI Neurofeedback for Treatment of Parkinson's Disease (NCT01867827). The aging study was supported by funding from the Medical Research Council, UK, *via* a Clinician Scientist Fellowship to Dr. Michael J O'Sullivan (G0701912). The working memory study was funded by Wellcome Trust New Investigator Award to Prof. Derek Jones (Grant Number: 502341), CM-B was supported by a research fellowship from the Alzheimer's Society and the BRACE Alzheimer's Charity (Grant Ref: 208).

## Conflict of Interest

The authors declare that the research was conducted in the absence of any commercial or financial relationships that could be construed as a potential conflict of interest.

## Publisher's Note

All claims expressed in this article are solely those of the authors and do not necessarily represent those of their affiliated organizations, or those of the publisher, the editors and the reviewers. Any product that may be evaluated in this article, or claim that may be made by its manufacturer, is not guaranteed or endorsed by the publisher.

## Supplementary Material

Supplementary Figure 1 The left and right dentato-rubro-thalamic tract (DRTT) on the axial and coronal views in T1 anatomical scan of representative subject. **(A)** shows the tracts passing through the dentate nucleus on left and right side, **(B)** shows the decussation of the tracts to the contralateral hemispheres, **(C)** shows the tracts passing through the thalamus and in this example passing onto the supplementary motor area and the primary motor cortex. **(D)** shows the two tracts in the coronal view.

Supplementary Figure 2 The reconstructed subthalamo-ponto-cerebellar tract (SPCT) in an axial view of a representative subject, **(A)** shows the left SPCT with the alternative regions of interest (ROIs) 1 and 2, **(B)** shows the left SPCT, **(C)** shows the right SPCT, and **(D)** shows both the right and the left SPCTs.

Supplementary Figure S3 Reconstruction of the middle cerebellar peduncle (MCP). The figure shows the AND region in green in a color-coded fiber orientation image **(A)** in an example dataset for reconstruction of the MCP. The MCP on a T1 image of a representative participant is shown in axial **(B)** and sagittal **(C)** views, respectively.

Supplementary Figure S4 Reconstruction of the superior cerebellar peduncle (SCP). The figure shows the AND region in green **(A)** and the NOT regions along with the reconstructed SCP in the color-coded fiber orientation image **(B)** and the reconstructed SCP on a T1 image **(C)** in an example dataset. Red NOT regions, ROI 1 and ROI 2, were drawn above and in front of the fornix, ROI 3 at the longitudinal fissure, and ROI 4 to cut fibers toward the brainstem.

Supplementary Figure S5 Reconstruction of the inferior cerebellar peduncle (ICP). The figure shows the first AND region **(A)**, second AND region **(B)**, the three retained NOT regions along with a reconstructed ICP tract in a color-coded fiber orientation image **(C)** and the reconstructed ICP in a T1 anatomical image **(D)** in an example dataset. Red NOT regions, ROI 1 and ROI 2, were drawn above and in front of the fornix and final ROI 3 at the longitudinal fissure.

Supplementary Figure S6 Reconstructed full and segmented DRTT and SPCT. The reconstructed DRTT with its cortical projections is shown in **(A)** and the segmented DRTT is shown in **(B)** along with the ROIs at the level of the thalamus and the dentate nucleus. The fully reconstructed SPCT with its cortical connections is shown in **(C)** and the segmented SPCT is shown in **(D)** along with the ROI at the level of the subthalamic nucleus.

Supplementary Table 1 Inter-operator reliability. Spatial dice coefficients across the tracts that were independently segmented by two operators are reported in column 1. Statistical comparison (paired *t*-test) between the size of the independently segmented tracts are reported in column 2. Pearson correlation coefficients between tract-averaged fractional anisotropy (FA) from the two independently segmented tracts are reported in column 3. L, Left; R, Right.

Supplementary Table 2 Principal component analysis on cerebellar tracts after Varimax rotation of the first three components. For each resulting component, the explained variance, associated eigenvalue, and the component loadings of all considered tracts are reported, as is the statistical comparison between patients (PD) and healthy controls (HC), as computed with a two-sample *t-*test. L, Left; R, Right.

Supplementary Table 3 Principal component analysis on cerebellar tracts after oblique rotation of the first three components. For each resulting component, the explained variance, associated eigenvalue, and the component loadings of all considered tracts are reported, as is the statistical comparison between patients (PD) and healthy controls (HC), as computed with a two-sample *t*-test. L, Left; R, Right.
