# Supplementary figures and images for: Investigating the Anatomy and Microstructure of the Dentato-rubro-thalamic and Subthalamo-ponto-cerebellar Tracts in Parkinson's Disease
Source: Front Neurol. 2022 Mar 24;13:793693. doi: 10.3389/fneur.2022.793693 (PMC8987292; doi:10.3389/fneur.2022.793693)

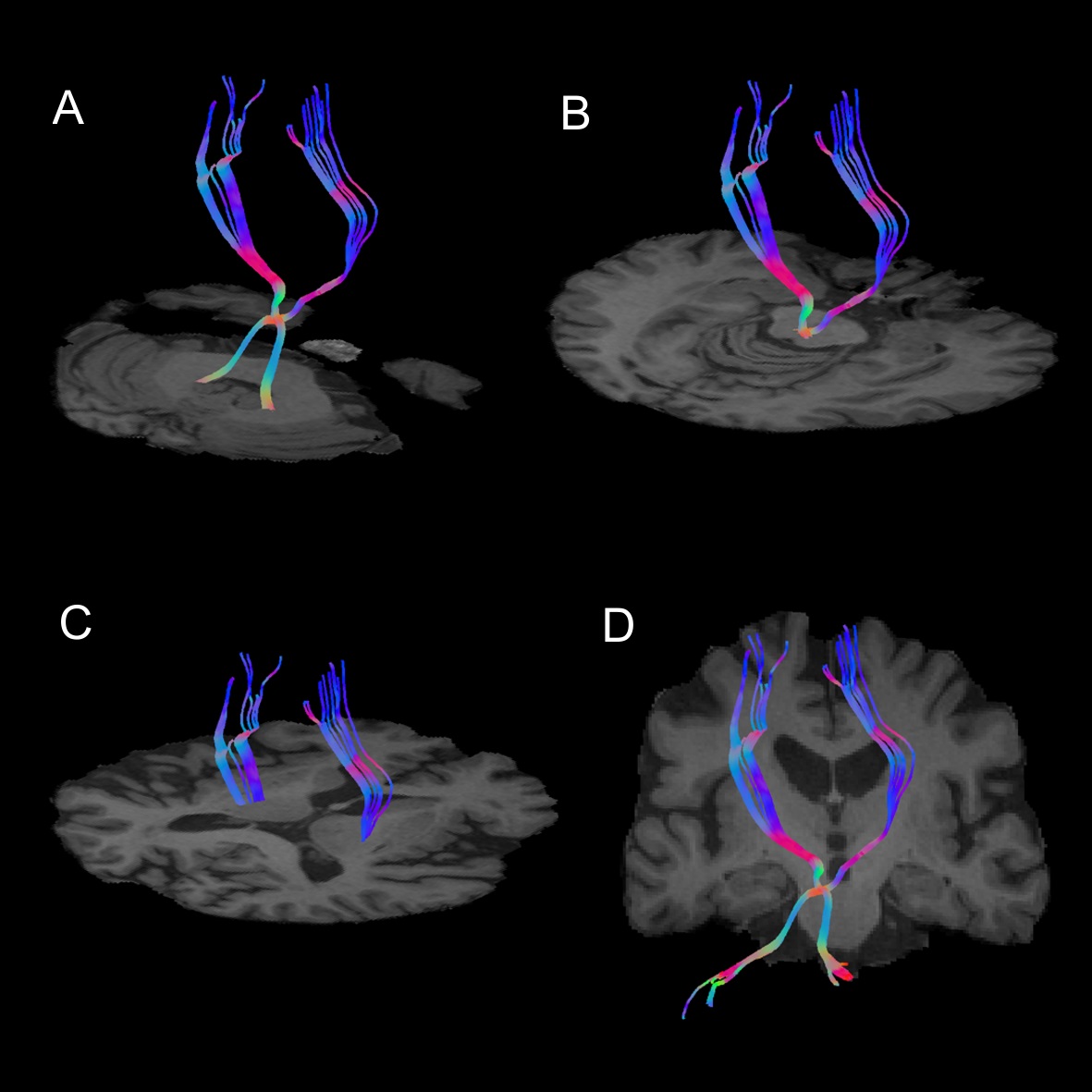

Supplement: Supplementary Figure 1 — The left and right dentato-rubro-thalamic tract (DRTT) on the axial and coronal views in T1 anatomical scan of representative subject. (A) shows the tracts passing through the dentate nucleus on left and right side, (B) shows the decussation of the tracts to the contralateral hemispheres, (C) shows the tracts passing through the thalamus and in this example passing onto the supplementary motor area and the primary motor cortex. (D) shows the two tracts in the coronal view. [file Image_1.JPEG]

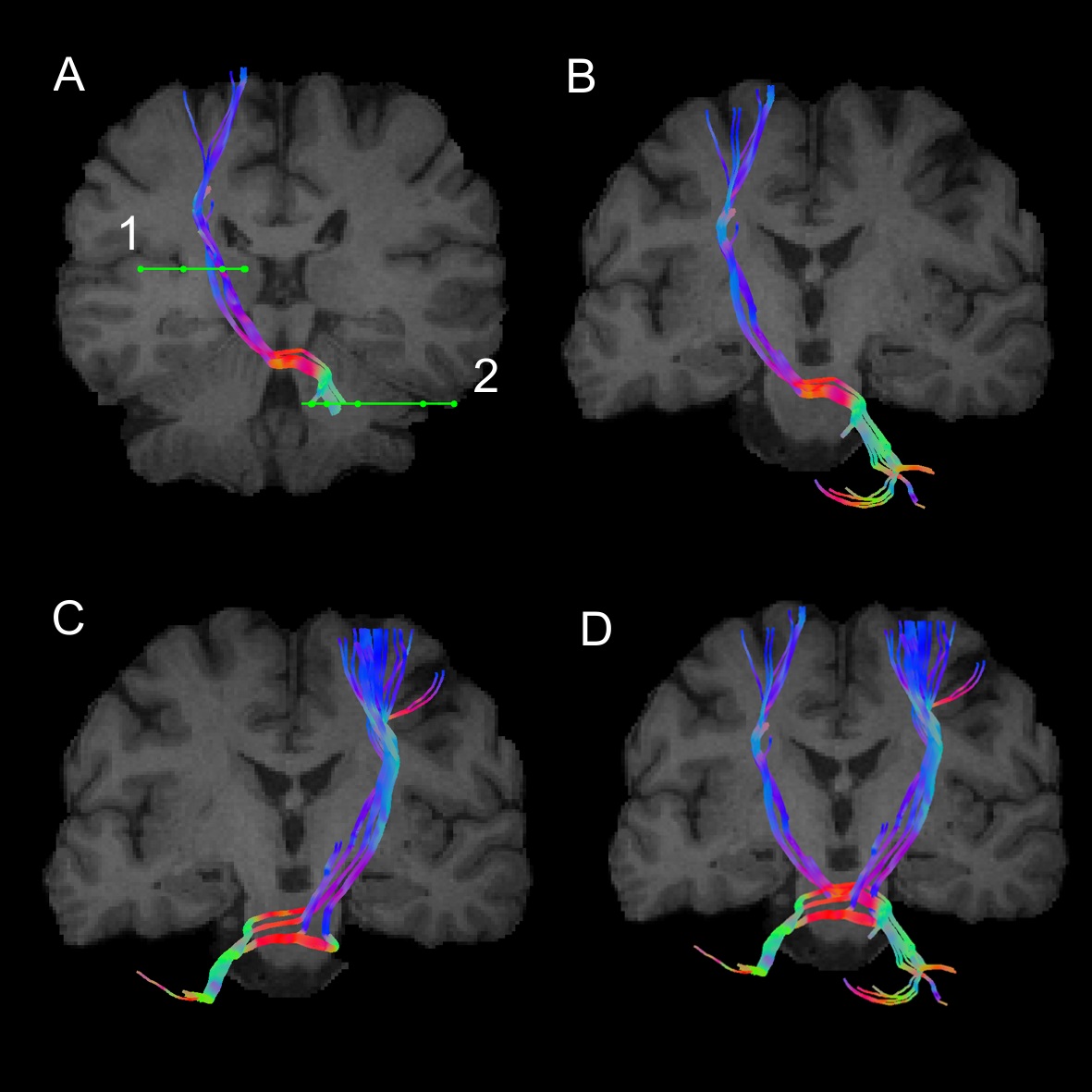

Supplement: Supplementary Figure 2 — The reconstructed subthalamo-ponto-cerebellar tract (SPCT) in an axial view of a representative subject, (A) shows the left SPCT with the alternative regions of interest (ROIs) 1 and 2, (B) shows the left SPCT, (C) shows the right SPCT, and (D) shows both the right and the left SPCTs. [file Image_2.JPEG]

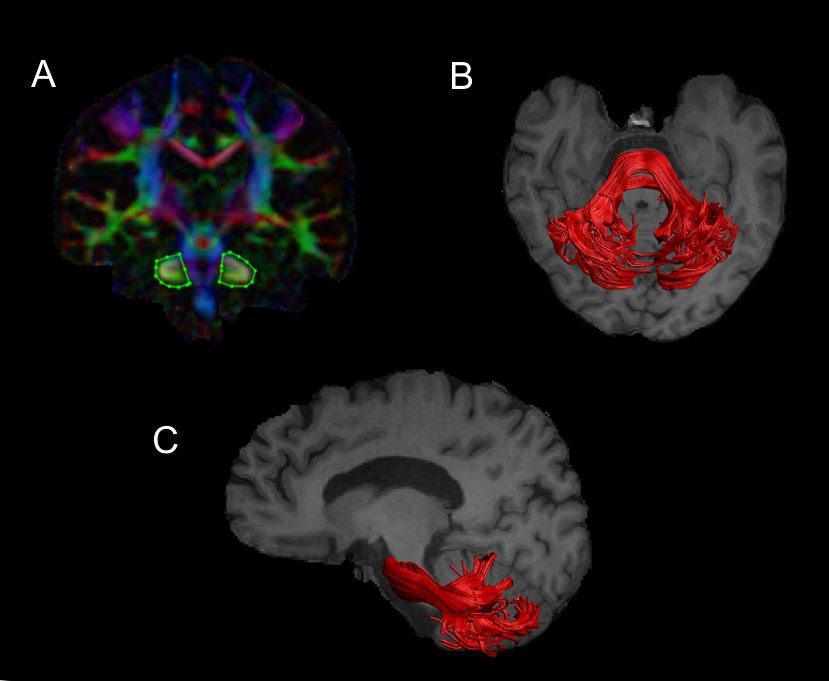

Supplement: Supplementary Figure S3 — Reconstruction of the middle cerebellar peduncle (MCP). The figure shows the AND region in green in a color-coded fiber orientation image (A) in an example dataset for reconstruction of the MCP. The MCP on a T1 image of a representative participant is shown in axial (B) and sagittal (C) views, respectively. [file Image_3.JPEG]

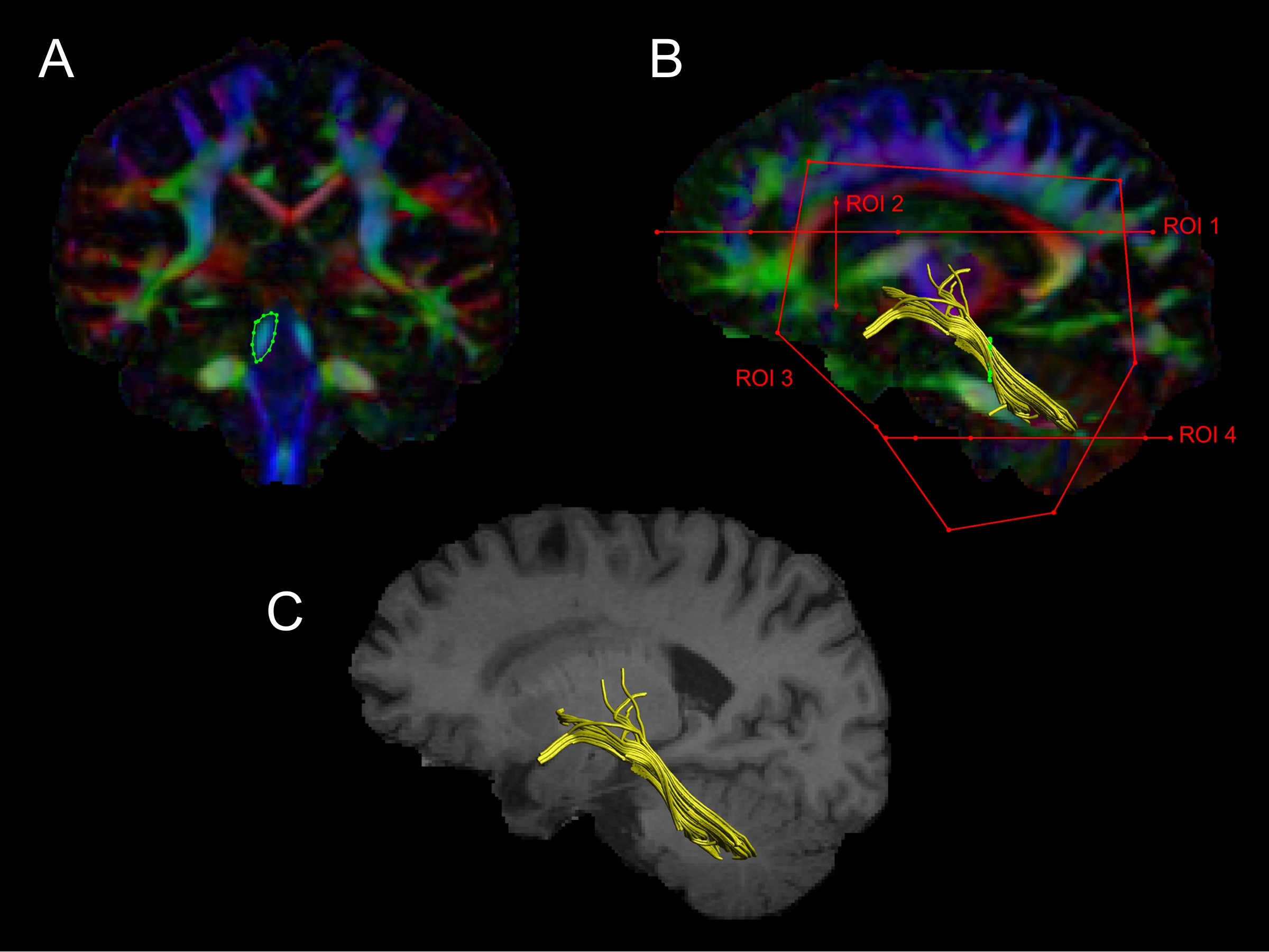

Supplement: Supplementary Figure S4 — Reconstruction of the superior cerebellar peduncle (SCP). The figure shows the AND region in green (A) and the NOT regions along with the reconstructed SCP in the color-coded fiber orientation image (B) and the reconstructed SCP on a T1 image (C) in an example dataset. Red NOT regions, ROI 1 and ROI 2, were drawn above and in front of the fornix, ROI 3 at the longitudinal fissure, and ROI 4 to cut fibers toward the brainstem. [file Image_4.JPEG]

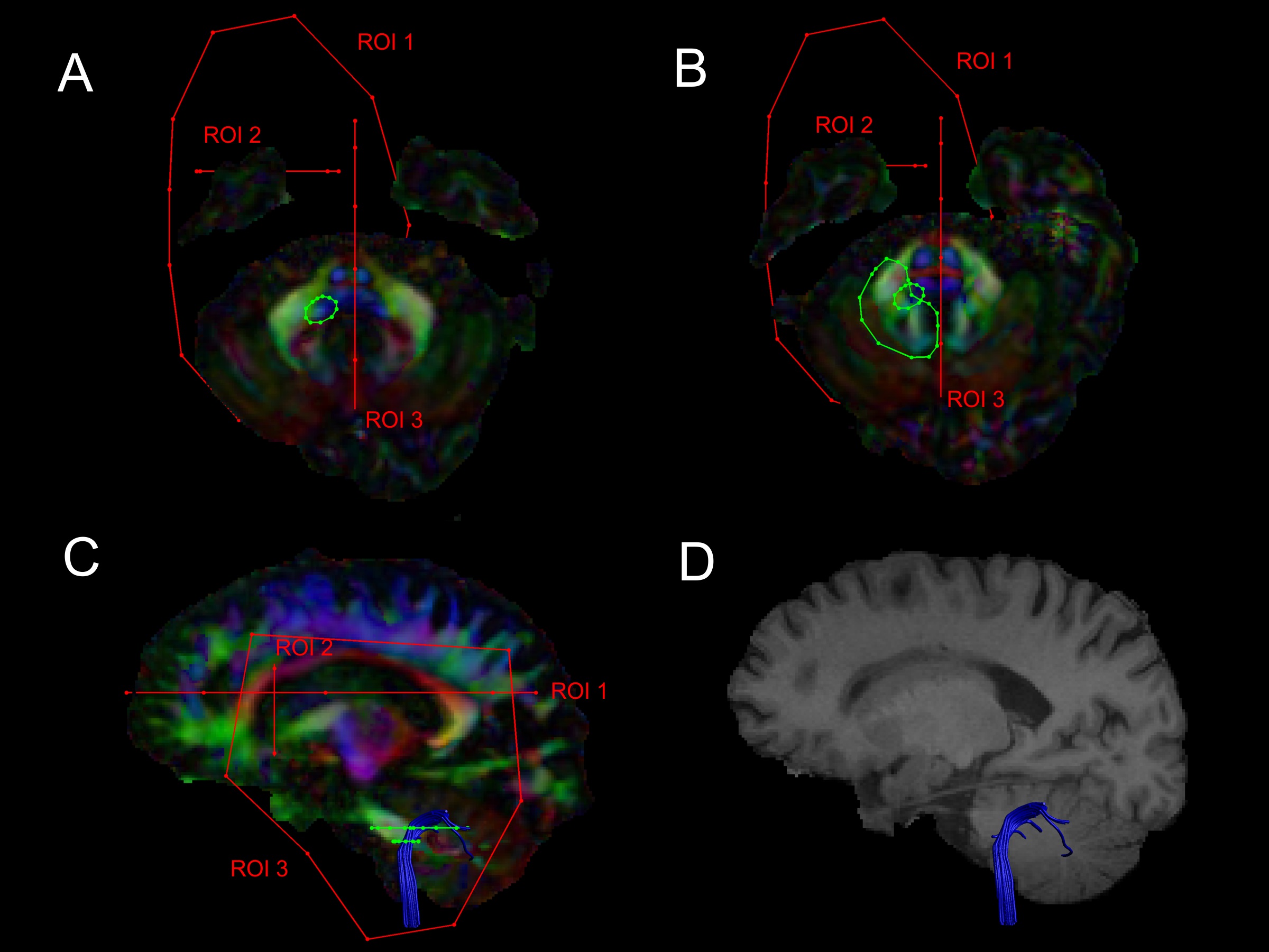

Supplement: Supplementary Figure S5 — Reconstruction of the inferior cerebellar peduncle (ICP). The figure shows the first AND region (A), second AND region (B), the three retained NOT regions along with a reconstructed ICP tract in a color-coded fiber orientation image (C) and the reconstructed ICP in a T1 anatomical image (D) in an example dataset. Red NOT regions, ROI 1 and ROI 2, were drawn above and in front of the fornix and final ROI 3 at the longitudinal fissure. [file Image_5.JPEG]

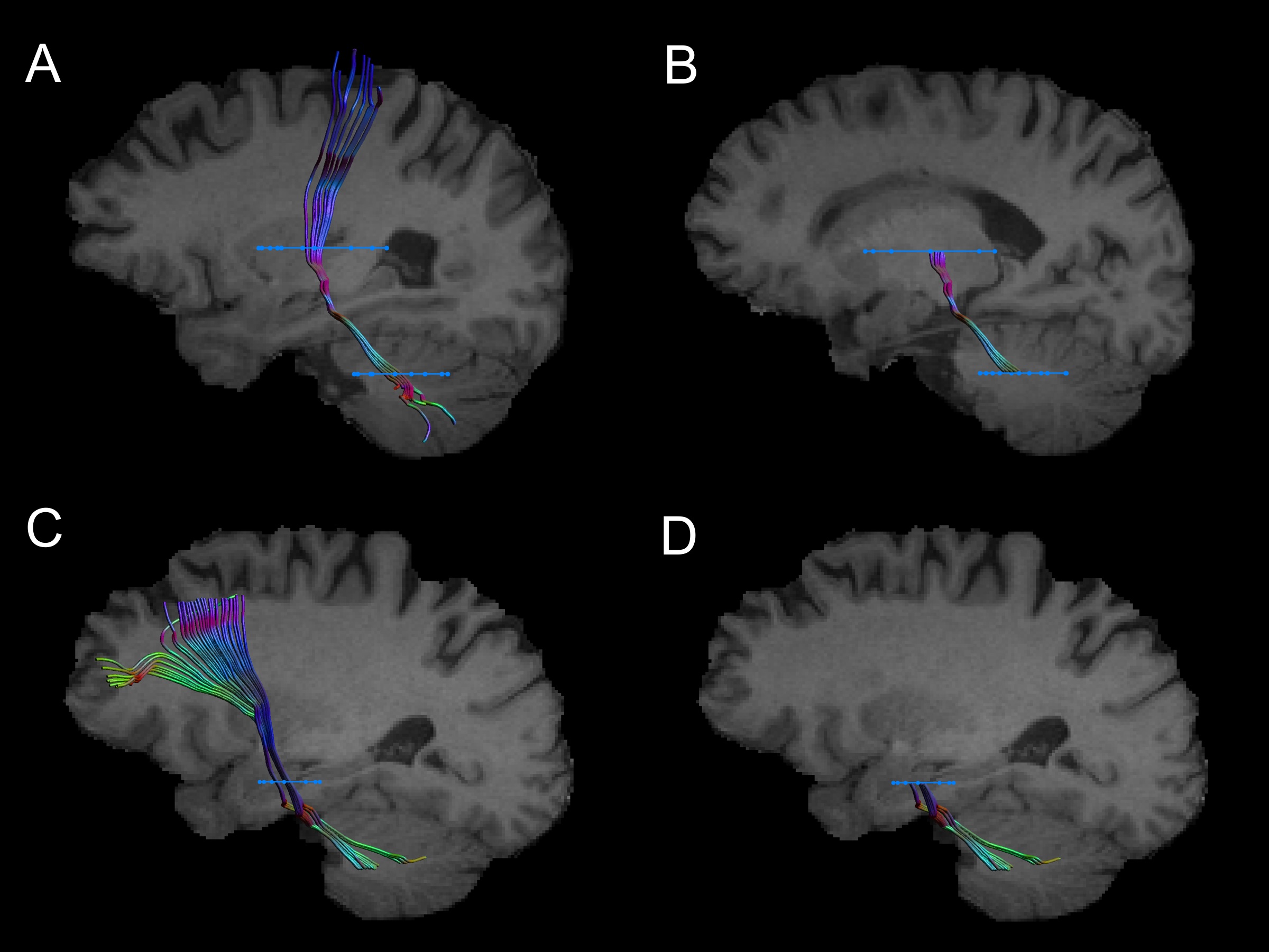

Supplement: Supplementary Figure S6 — Reconstructed full and segmented DRTT and SPCT. The reconstructed DRTT with its cortical projections is shown in (A) and the segmented DRTT is shown in (B) along with the ROIs at the level of the thalamus and the dentate nucleus. The fully reconstructed SPCT with its cortical connections is shown in (C) and the segmented SPCT is shown in (D) along with the ROI at the level of the subthalamic nucleus. [file Image_6.JPEG]
