# Supplementary material for: Investigating the Anatomy and Microstructure of the Dentato-rubro-thalamic and Subthalamo-ponto-cerebellar Tracts in Parkinson's Disease
Source: Front Neurol. 2022 Mar 24;13:793693. doi: 10.3389/fneur.2022.793693 (PMC8987292; doi:10.3389/fneur.2022.793693)
Supplement: Supplementary Table 1 — Inter-operator reliability. Spatial dice coefficients across the tracts that were independently segmented by two operators are reported in column 1. Statistical comparison (paired t-test) between the size of the independently segmented tracts are reported in column 2. Pearson correlation coefficients between tract-averaged fractional anisotropy (FA) from the two independently segmented tracts are reported in column 3. L, Left; R, Right. [file Data_Sheet_1.PDF]

Supplementary Table 1 Inter-operator reliability. Spatial dice coefficients across the tracts that were independently segmented by two operators are reported in column 1. Statistical comparison (paired *t*-test) between the size of the independently segmented tracts are reported in column 2. Pearson correlation coefficients between tract-averaged fractional anisotropy (FA) from the two independently segmented tracts are reported in column 3. L = Left. R = Right.

	Mean ± std Inter-operator Dice coefficients in %	Between-operater comparison of segmented tract size	Inter-operator correlation of tract-FA
L DRTT	64 ± 27	$t(9) = -1.89$; $p = .092$	$r = 0.82$, $p = 0.0238$
R DRTT	71 ± 32	$t(6) = -2.74$; $p = .034$	$r = 0.997$, $p = 0.0033$
L SPCT	45 ± 37	$t(8) = -1.81$; $p = .108$	$r = 0.28$, $p = 0.5051$
R SPCT	52 ± 43	$t(8) = -1.02$; $p = .336$	$r = -0.24$, $p = 0.6996$
MCP	93 ± 5	$t(14) = -0.29$; $p = .775$	$r = 0.98$, $p < .0001$
L ICP	58 ± 22	$t(14) = -1.20$; $p = .069$	$r = 0.87$, $p = 0.0009$
R ICP	67 ± 15	$t(14) = -2.80$; $p = .014$	$r = 0.93$, $p = 0.0001$
L SCP	66 ± 15	$t(14) = -2.50$; $p = .025$	$r = 0.58$, $p = 0.08$
R SCP	62 ± 13	$t(14) = -3.03$; $p = .009$	$r = 0.90$, $p = 0.0003$

Supplementary Table 2: Principal component analysis on cerebellar tracts after Varimax rotation of the first three components. For each resulting component, the explained variance, associated eigenvalue, and the component loadings of all considered tracts are reported, as is the statistical comparison between patients (PD) and healthy controls (HC), as computed with a two-sample *t*-test. L = Left. R = Right.

#	Variance	Eigenvalue	Tract loadings	PD vs HC
1	25%	2.22	L DRTT: 0.70, R DRTT: 0.47, L SPCT: -0.38, R SPCT: 0.10, MCP: 0.24, L ICP: 0.24, R ICP: 0.38, L SCP: 0.69, R SCP: 0.78	$t(40.6) = 3.52$, $p = 0.001$
2	22%	2.02	L DRTT: 0.04, R DRTT: 0.55, L SPCT: 0.58, R SPCT: 0.80, MCP: 0.78, L ICP: 0.03, R ICP: 0.15, L SCP: 0.10, R SCP: 0.30	not retained
3	21%	1.85	L DRTT: 0.11, R DRTT: 0.17, L SPCT: 0.48, R SPCT: -0.08, MCP: 0.18, L ICP: 0.89, R ICP: 0.74, L SCP: 0.41, R SCP: 0.22	not retained

Supplementary Table 3: Principal component analysis on cerebellar tracts after oblique rotation of the first three components. For each resulting component, the explained variance, associated eigenvalue, and the component loadings of all considered tracts are reported, as is the statistical comparison between patients (PD) and healthy controls (HC), as computed with a two-sample *t*-test. L = Left. R = Right.

#	Variance	Eigenvalue	Tract loadings	PD vs HC
1	19%	1.72	L DRTT: 0.65, R DRTT: 0.31, L SPCT: -0.60, R SPCT: -0.02, MCP: 0.05, L ICP: -0.02, R ICP: 0.14, L SCP: 0.54, R SCP: 0.65	$t(42.4) = 3.51$, $p = 0.001$
2	24%	2.20	L DRTT: 0.09, R DRTT: 0.58, L SPCT: 0.48, R SPCT: 0.86, MCP: 0.81, L ICP: -0.11, R ICP: 0.05, L SCP: 0.09, R SCP: 0.35	not retained
3	24%	2.16	L DRTT: 0.15, R DRTT: 0.11, L SPCT: 0.39, R SPCT: -0.22, MCP: 0.07, L ICP: 0.95, R ICP: 0.78, L SCP: 0.46, R SCP: 0.22	not retained
